# Circulating Exosomes Are Strongly Involved in SARS-CoV-2 Infection

**DOI:** 10.3389/fmolb.2021.632290

**Published:** 2021-02-22

**Authors:** Elettra Barberis, Virginia V. Vanella, Marco Falasca, Valeria Caneapero, Giuseppe Cappellano, Davide Raineri, Marco Ghirimoldi, Veronica De Giorgis, Chiara Puricelli, Rosanna Vaschetto, Pier Paolo Sainaghi, Stefania Bruno, Antonio Sica, Umberto Dianzani, Roberta Rolla, Annalisa Chiocchetti, Vincenzo Cantaluppi, Gianluca Baldanzi, Emilio Marengo, Marcello Manfredi

**Affiliations:** ^1^ Department of Translational Medicine, University of Piemonte Orientale, Novara, Italy; ^2^ Center for Translational Research on Autoimmune and Allergic Diseases, University of Piemonte Orientale, Novara, Italy; ^3^ Metabolic Signalling Group, Curtin Medical School, Curtin University, Perth, WA, Australia; ^4^ Department of Health Sciences, University of Piemonte Orientale, Novara, Italy; ^5^ Internal and Emergency Medicine Departments, Department of Translational Medicine, University of Piemonte Orientale, Novara, Italy; ^6^ Azienda Ospedaliero-Universitaria "Maggiore della Carità", Novara, Italy; ^7^ Città della Salute e della Scienza and Molecular Biotechnology Center, Torino, Italy; ^8^ Department of Sciences and Technological Innovation, University of Piemonte Orientale, Alessandria, Italy; ^9^ ISALIT, Novara, Italy; ^10^ Department of Pharmaceutical Sciences, University of Piemonte Orientale, Novara, Italy; ^11^ Humanitas Clinical and Research Center, IRCCS, Milan, Italy

**Keywords:** SARS-CoV-2, plasma exosomes, host-response, biomarkers, proteomics

## Abstract

Knowledge of the host response to the novel coronavirus SARS-CoV-2 remains limited, hindering the understanding of COVID-19 pathogenesis and the development of therapeutic strategies. During the course of a viral infection, host cells release exosomes and other extracellular vesicles carrying viral and host components that can modulate the immune response. The present study used a shotgun proteomic approach to map the host circulating exosomes’ response to SARS-CoV-2 infection. We investigated how SARS-CoV-2 infection modulates exosome content, exosomes’ involvement in disease progression, and the potential use of plasma exosomes as biomarkers of disease severity. A proteomic analysis of patient-derived exosomes identified several molecules involved in the immune response, inflammation, and activation of the coagulation and complement pathways, which are the main mechanisms of COVID-19–associated tissue damage and multiple organ dysfunctions. In addition, several potential biomarkers—such as fibrinogen, fibronectin, complement C1r subcomponent and serum amyloid P-component—were shown to have a diagnostic feature presenting an area under the curve (AUC) of almost 1. Proteins correlating with disease severity were also detected. Moreover, for the first time, we identified the presence of SARS-CoV-2 RNA in the exosomal cargo, which suggests that the virus might use the endocytosis route to spread infection. Our findings indicate circulating exosomes’ significant contribution to several processes—such as inflammation, coagulation, and immunomodulation—during SARS-CoV-2 infection. The study’s data are available via ProteomeXchange with the identifier PXD021144.

## 1 Introduction

The novel coronavirus disease (COVID-19) is now responsible for over one million deaths worldwide (COVID-19 Data in Motion, 2020). The disease’s clinical presentation following infection is challenging, ranging from asymptomatic patients or mild-to-moderate respiratory infections in individuals with atypical pneumonia. Peculiar features of acute respiratory distress syndrome (ARDS) require admission to intensive care units and mechanical ventilation. Although respiratory failure is the most common clinical presentation of severe COVID-19 cases, dysfunctions of other organs—including the kidneys and heart—have also been reported (Wu et al., 2020). The SARS-CoV-2 virus can infect multiple cell types, including lung epithelial cells, lymphocytes, and other types of leukocytes subsets (Bost et al., 2020). Moreover, Varga et al., (2020) demonstrated that SARS-CoV-2 could enter the endothelial cells of several organs; notably, endothelial infection is responsible for viral dissemination and triggering of the coagulation and complement cascades, which are key elements in the host’s thromboinflammatory response and the development of multiple organ failures (Guglielmetti et al., 2020; Noris et al., 2020). In our previous work we used a comprehensive untargeted metabolomic and lipidomic approach to capture the host response to SARS-CoV-2 infection, providing evidence that lipids and metabolic dysfunction are strongly involved in COVID-19 (Barberis et al.,2020).

Considering the wide clinical presentation of COVID-19, the scientific community still needs to improve its knowledge about optimal prevention, early diagnosis, and adequate therapeutic options. In this scenario, extracellular vesicles (EVs) may represent an important tool for COVID-19 management. EVs are micro-particles released from different types of activated cells, and they play a key role in the mechanisms of intercellular cross-talk through the direct transfer to target cells of proteins, receptors, lipids, organelles, and genetic materials such as mRNA and microRNA. EVs are present in different body fluids—including plasma, urine, saliva, and liquor—increasingly offering an opportunity to apply OMIC technologies in order to study the host response to viral infections and for biomarker discovery. Proteomics technology has been widely used to characterize and study EVs (Bandu et al., 2019; Choi et al., 2015; Xu et al., 2020; Adamo et al., 2019; Bonafede et al., 2019). EVs can be classified into three main families: 1) exosomes that originate from multivesicular bodies, ranging in size from 30 to 150 nm; 2) microvesicles or shedding vesicles released by cells through a membrane-sorting process promoted by enzymes such as flippase, floppase, and scramblase and characterized by a size larger than exosomes (150–1,000 nm); and 3) apoptotic bodies that are larger than exosomes and microvesicles and that are released by cells undergoing programmed cell death (Camussi et al., 2013).

In recent years, EVs isolation and characterization protocols have considerably improved (Théry et al., 2018), offering new opportunities to study their roles both as biomarkers and as mediators of several human diseases (Maione et al., 2020). Based on these considerations, this study aimed to characterize—via proteomic analysis—any alterations of exosome content upon SARS-CoV-2 infection as well as the potential use of plasma exosomes as biomarkers to monitor SARS-CoV-2 infection severity. Moreover, the identification of several molecules involved in processes such as immune response, and inflammation, the activation of coagulation and complement pathways could link circulating exosomes to COVID-19–associated tissue damage and multiple organ dysfunctions.

## 2 Results

### 2.1 Exosomes Incorporate SARS-CoV-2 RNA

The presence of viral RNA in the exosome cargo was investigated using reverse transcription-droplet digital polymerase chain reaction (RT-ddPCR). RT-ddPCR enables a significant gain in dynamic range while decreasing the cost of analysis. In addition, it is more sensitive than qPCR, and it provides more accurate data—especially at low target copy numbers (Rački et al., 2014). Analysis of exosome content purified from critical and non-critical patients revealed the presence of SARS-CoV-2 RNA in the exosomal cargo. We found viral material that ranges from 15 to 88 copies/10 µl with no significant differences between the two groups. No viral material was detected in healthy subjects.

### 2.2 Proteomic Analysis of Plasma-Derived Exosomes From COVID-19 Patients

Untargeted proteomic analysis was performed on plasma-derived exosomes from 17 SARS-CoV-2 positive patients and seven healthy controls. The patients enrolled in this study resided in Northern Italy, which was the COVID-19 pandemic’s Italian epicenter.

We divided our patient group in two cohorts: critical (patients with respiratory failure who were admitted to intensive care units, requiring mechanical ventilation), and non-critical (all other patients, with mild to severe respiratory failure, requiring oxygen supplementation but neither invasive nor noninvasive mechanical ventilation). Out of 17 patients, seven patients were in critical condition, and 10 were in non-critical condition. Critical COVID-19 patients’ blood levels of white blood cells (WBCs) and eosinophil were significantly higher than non-critical patients. On the contrary, we found a slight increase in red blood cells and lymphocyte counts among non-critical COVID-19 patients (Supplementary Table S1).

**FIGURE 1:**
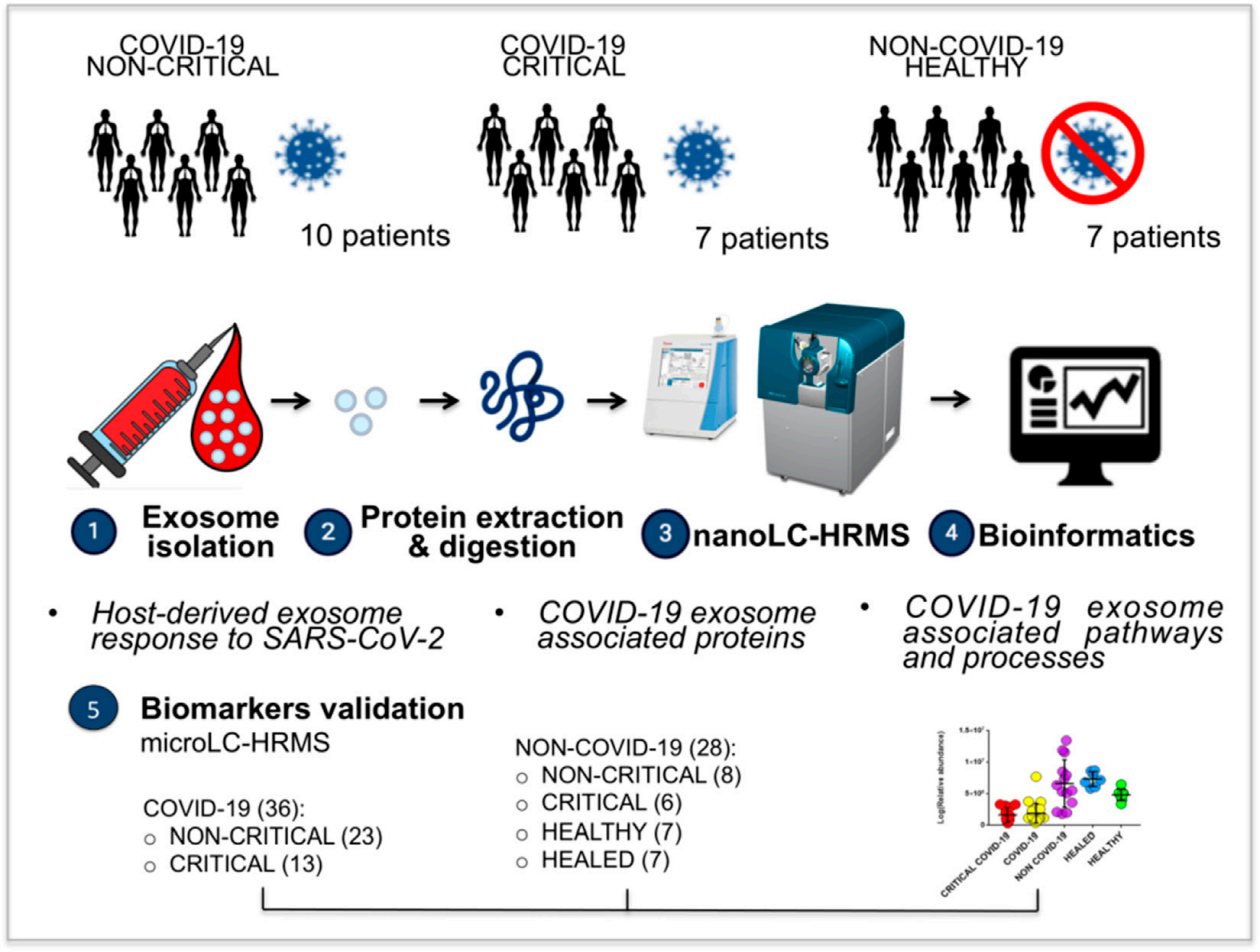
Overview of this study’s experimental design: plasma exosomes from 10 non-critical COVID-19 patients, seven critical COVID-19 patients, and seven healthy subjects were isolated using a commercial kit. The exosomes’ protein content was analyzed using proteomics analysis (nanoLC-HRMS), and the identified and modulated proteins were elaborated with bioinformatics in order to identify the host-derived exosome response to SARS-CoV-2 and its associated pathways. The analysis suggested the presence of new biomarkers. The validation of potential exosomal biomarkers was performed on an external cohort of patients using a proteomics approach on a microLC-HRMS. 36 COVID-19 patients, including non-critical (23) and critical (13) subjects, and on 28 non-COVID-19 patients, including 6 critical patients, 8 non-critical patients, 7 healthy subjects and 7 healed COVID-19 subjects were analyzed.

Figure 1 provides an overview of this study’s experimental design. A brief description follows here. Exosomes were isolated from plasma. The purification of the exosomes was subjected to several control analyses. Nanosight and Western blotting were employed to characterize the quality of the method of purification; these analyses, which were performed only on healthy subjects for safety reasons, confirmed the isolation of exosomes with a size ranging from 30 to 100 nm (peak 37.70 ± 3 nm) and a concentration of 3x10^11^ particles/ml (Supplementary Figure S1). In addition, the typical exosomal markers CD9 and CD63 were detected, confirming these vesicles as exosomes.

Exosomal proteins were then extracted, digested, and analyzed using a nano-liquid chromatography/tandem mass spectrometry (nanoLC-MS/MS). The results were elaborated using bioinformatics tools to highlight the main functions and pathways associated with the host response to SARS-CoV-2 infection.

The validation of potential biomarkers was then performed using a microLC-MS/MS on 36 COVID-19 patients, including non-critical (23) and critical (13) subjects, and on 39 non-COVID-19 patients, including 6 critical patients, 8 non-critical patients, 7 healthy subjects and 7 healed COVID-19 subjects.

### 2.3 Circulating Exosomes in COVID-19 Patients Have a Specific Proteomic Signature

To identify the proteins potentially involved in the development of SARS-CoV-2 infection and in any immunomodulatory functions mediated by circulating exosomes, plasma exosomes from COVID-19 patients with varying disease severity were analyzed using shotgun proteomic analysis.

**FIGURE 2:**
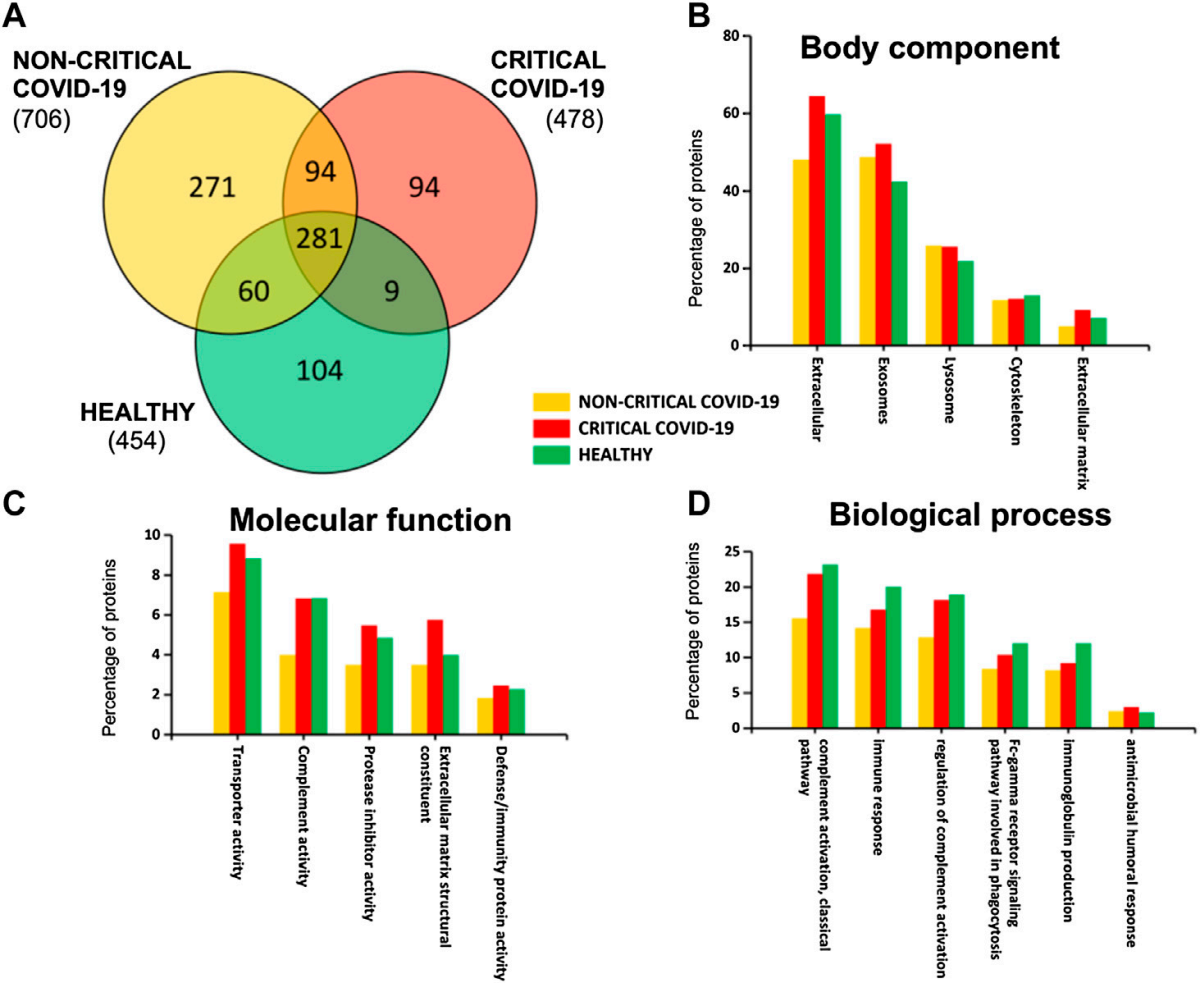
Plasma-exosomes protein content. A Venn diagram **(A)** of identified proteins in critical and non-critical COVID-19 patients and in healthy subjects. Gene ontology classification of identified proteins based on body component **(B)**, molecular function **(C)**, and biological process **(D)** was also used to assess the functions associated to identified proteins.

A total of 913 different proteins were identified in plasma exosomes; among them, 281 were found in critical, non-critical, and healthy subjects, as Figure 2 shows. Interestingly, non-critical patients are characterized by the presence of a higher number of proteins (706), while a similar number of proteins were identified in critical patients (478) and healthy subjects (454) (Supplementary Table S2).

By analyzing exosomal cargo, we found that it was mainly characterized by the presence of extracellular, exosomal, lysosome, and cytoskeleton proteins (Figure 2B). Moreover, these proteins are involved in transport activity, complement activity, protease inhibitor activity, extracellular matrix structural constituents, and defense/immunity activity (Figure 2C). Interestingly, some proteins are associated with immune response and coagulation (complement activity, immune response, regulation of complement activation, Fc-gamma receptor signaling pathway, immunoglobulin production, and antimicrobial humoral response), as Figure 2D shows.

**FIGURE 3:**
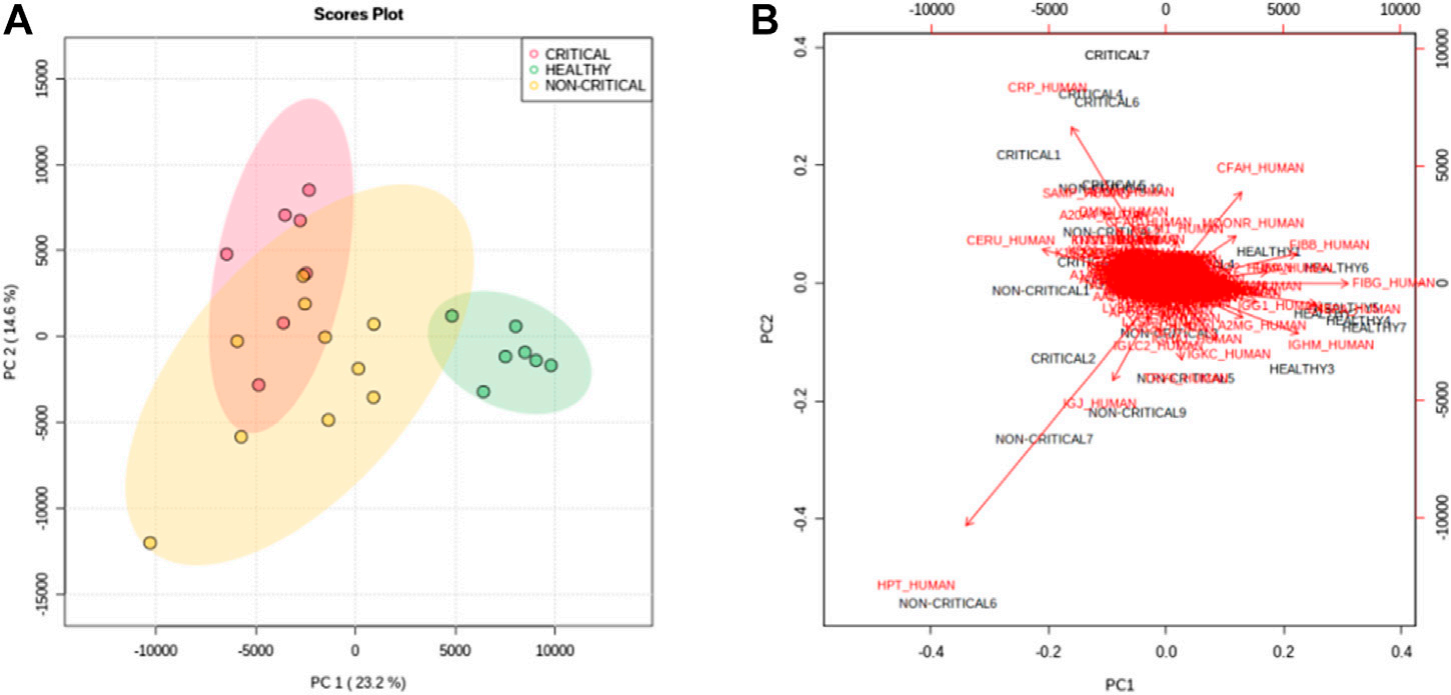
Principal component analysis. The score plot **(A)** of PC1 and PC2 shows a clear separation of healthy subjects (green dots) from critical COVID-19 patients (red dots) and non-critical (yellow dots) COVID-19 patients. **(B)** The bi-plot of the scores (black) and loading (red) of PC1 and PC2 reported the correlation between the severity of the disease and the expression of proteins.

To assess overall differences between exosomes from COVID-19 patients and healthy subjects, protein abundances were analyzed using multivariate statistical analysis. Principal component analysis (PCA)—in particular, the first and second principal components—clearly separated the samples according to the groups. The first component explained the differences between COVID-19 samples (red and yellow dots) and non-COVID-19 samples (green dots), while the second component mainly explained the differences between in disease severity (Figure 3A). Figure 3B reports a bi-plot of the scores and individual proteins’ loading information. The plot reports the proteins that are “driving the separation” between the patient groups. For example, interestingly, CRP protein (CRP_HUMAN) was able to discriminate positive patients from non-positive patients but also critical patients from non-critical patients.

**FIGURE 4:**
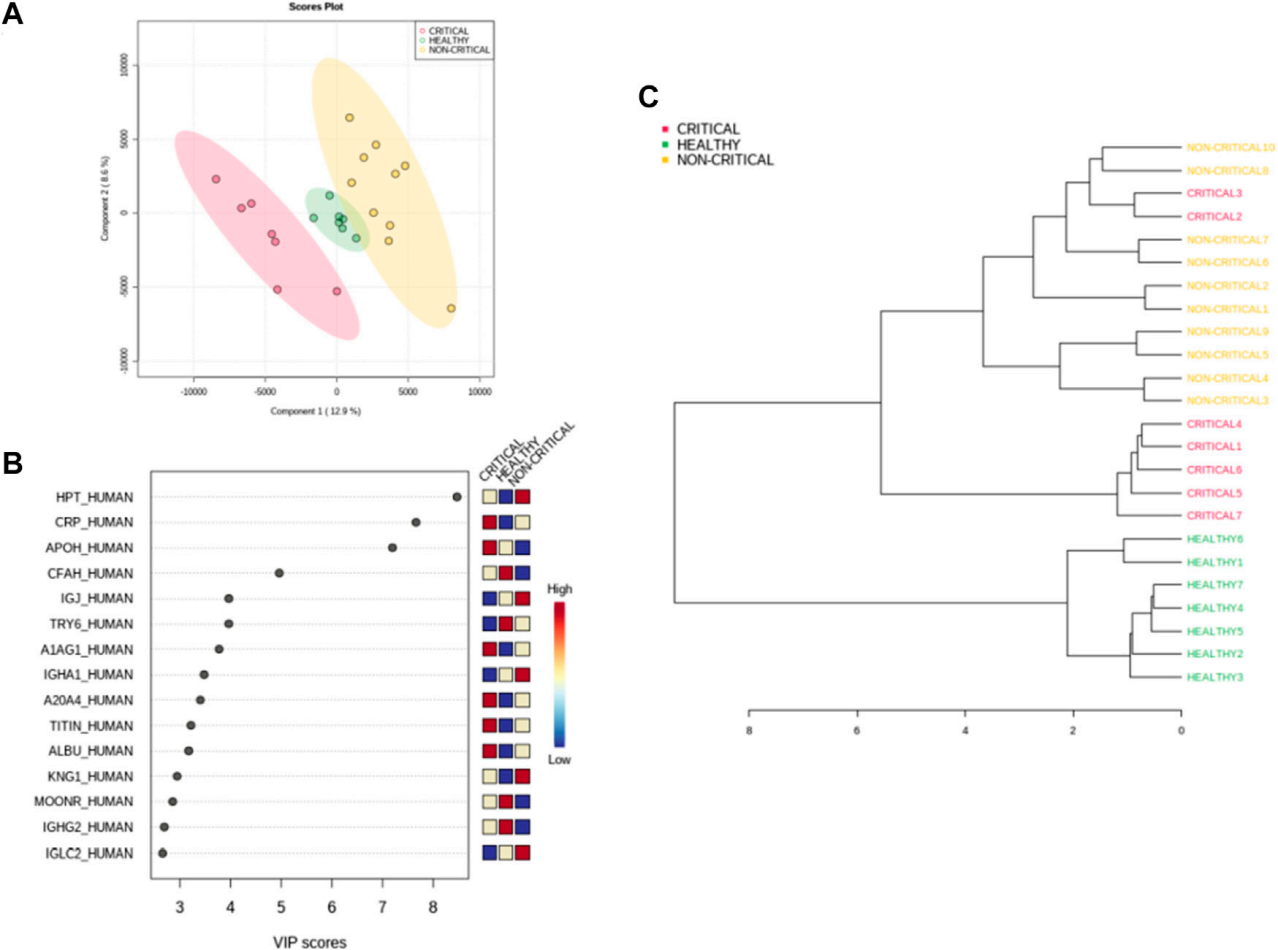
Modulated exosomal proteins in SARS-CoV-2 infection. Volcano plots of quantified proteins **(A and B)** reporting *p*-value and fold change. A total of 157 and 97 proteins were modulated in critical **(A)** and non-critical **(B)** COVID-19 patients, respectively (*p*-value < 0.05 and fold change > 1.3). Hierarchical heat maps of quantified proteins **(C)** highlighting the three clusters of samples, with critical COVID-19 patients in red, non-critical COVID-19 patients in yellow, and healthy subjects in green.

The samples were also classified using a supervised partial least square discriminant analysis (PLS-DA), which was exploited to achieve maximum separation between the two patient groups (Figure 4A). The most predictive or discriminative features that are potentially useful in helping sample classification were also determined through the VIP (variable of importance in projection) score. The VIP score summarized the most prominent proteins contributing to the observed phenotypic variations in the COVID-19 plasma exosomes (Figure 4B). Proteomics differences between the three groups of samples were mostly due to haptoglobin (HPT_HUMAN), C-reactive protein (CRP_HUMAN), Beta-2-glycoprotein 1 (APOH_HUMAN), and Complement factor H (CFAH_HUMAN). The dendrogram of hierarchical clustering of protein abundance in exosomes from critical patients (red), non-critical patients (yellow), and healthy subjects (green) confirmed that exosomal proteins correlated with the SARS-CoV-2 infection and with the disease’s severity (Figure 4C).

### 2.4 Exosomal Proteins are Strongly Involved in the Host Response to SARS-CoV-2

**FIGURE 5:**
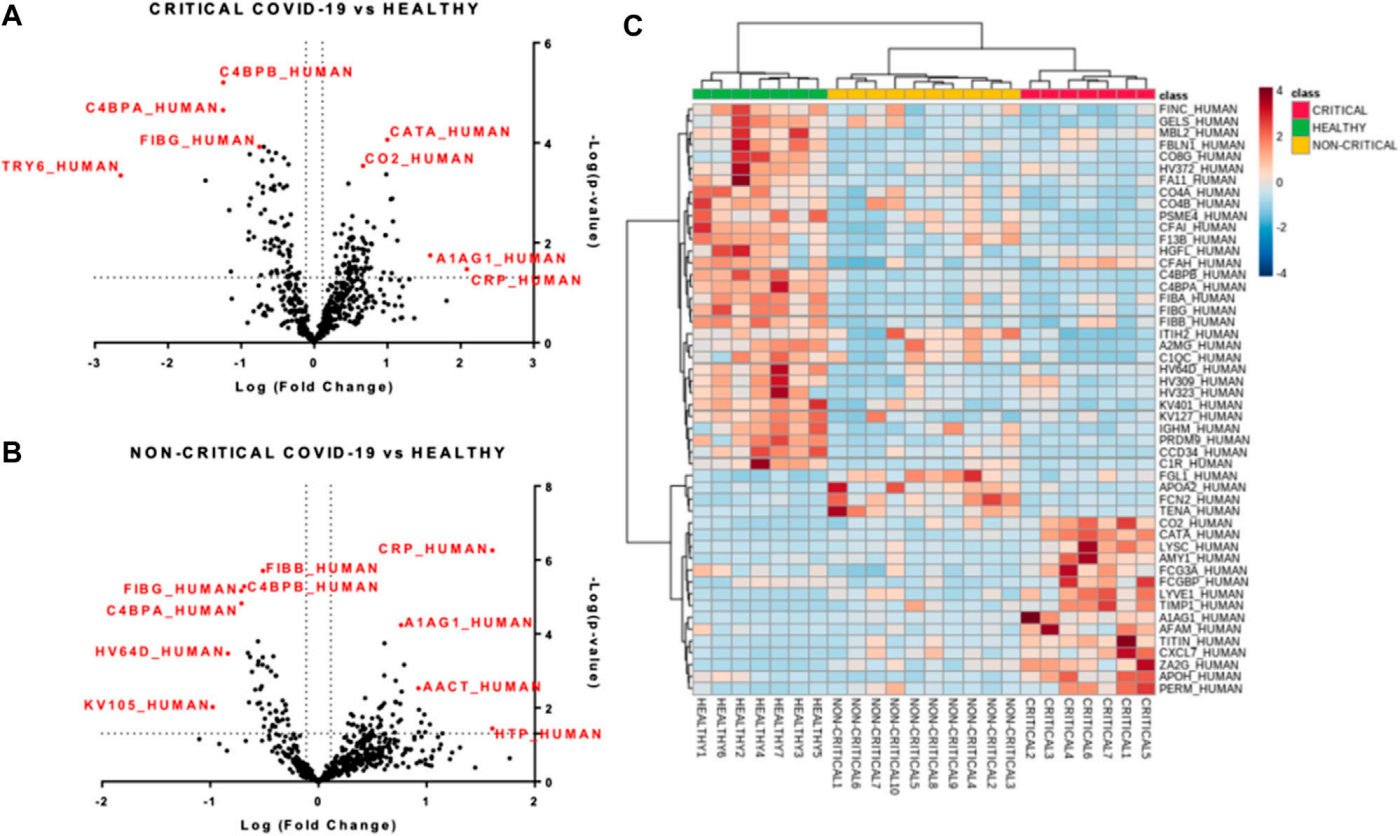
Modulated exosomal proteins in SARS-CoV-2 infection. Volcano plots of quantified proteins (A and B) reporting *p*-value and fold change. A total of 157 and 97 proteins were modulated in critical **(A)** and non-critical **(B)** COVID-19 patients, respectively (*p*-value < 0.05 and fold change > 1.3). Hierarchical heat maps of quantified proteins **(C)** highlighting the three clusters of samples, with critical COVID-19 patients in red, non-critical COVID-19 patients in yellow, and healthy subjects in green.

Next, we performed a univariate analysis of quantified proteins using the relative abundance of 518 proteins quantified in all samples. A total of 157 proteins were modulated in exosomes from critical COVID-19 patients compared to healthy subjects, while 97 proteins were regulated in non-critical patients (*p*-value < 0.05, fold change > 1.3). Volcano plots (Figure 5A and Figure 5B) showed the most significant differences among proteins and the positive or negative fold-changes in exosomes from critical and non-critical COVID-19 patients compared to the healthy group. To summarize the univariate results, we used a heat map (Figure 5C) to display the fold changes of the top modulated proteins. This heat map allowed visualization of the three clusters of samples and different protein levels. The complete list of modulated proteins is reported in Supplementary Tables S1, S2. The top regulated proteins in both critical and non-critical patients, compared to healthy subjects, mainly included inflammatory, immune-response, and coagulation proteins. In critical patients, C-reactive protein (122-fold), alpha-1-acid glycoprotein 1 (38-fold), lysozyme C (13-fold), titin (12-fold), and zinc-alpha-2-glycoprotein (12-fold) were up-regulated while putative trypsin-6 (31-fold), coiled-coil domain-containing protein 34 (18-fold), C4b-binding protein alpha chain (18-fold), C4b-binding protein beta chain (15-fold), and pre-mRNA-processing factor 19 (14-fold) were down-regulated. Among non-critical COVID-19 patients, the top five up-regulated proteins were haptoglobin (41-fold), C-reactive protein (40-fold), trypsin-3 (14-fold), adenomatous polyposis coli protein (11-fold), and hyaluronan-binding protein 2 (10-fold) while immunoglobulin kappa variables 1–5 (10-fold), immunoglobulin heavy variables 3–64D (7-fold), fibrinogen gamma chain (5-fold), C4b-binding protein alpha chain (5-fold), and C4b-binding protein beta chain (5-fold) were under-expressed.

### 2.5 Circulating Exosomes in COVID-19 Patients may Modulate Immune Response, Inflammation, and Coagulation Pathways

**FIGURE 6:**
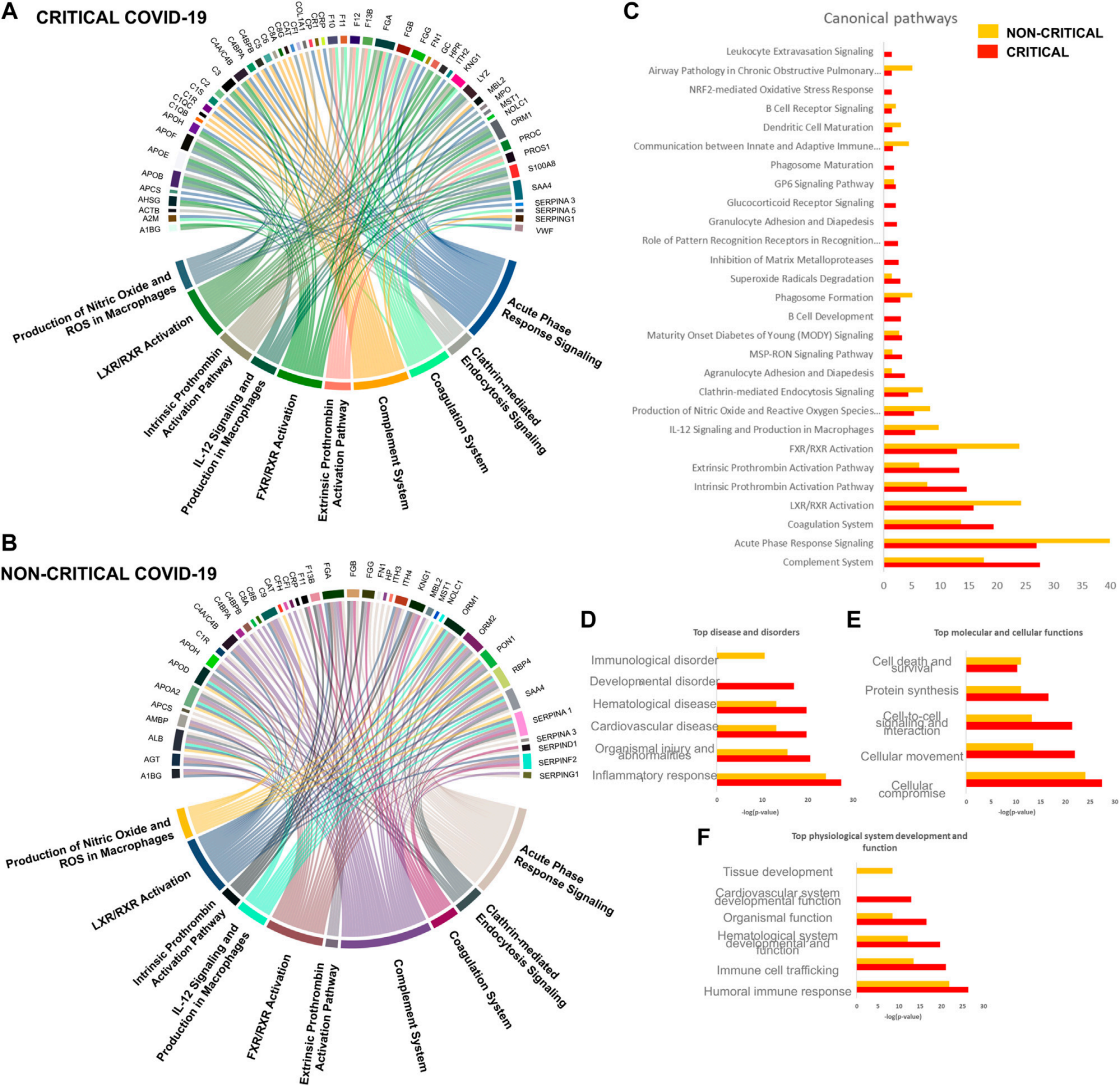
Host-derived exosome response to SARS-CoV-2 infection. Circulating exosomes are characterized by proteins and pathways involved in inflammation, immune response, and coagulation.

To obtain a global overview of the exosome proteomic response in COVID-19, modulated proteins were analyzed with bioinformatics tools. Ingenuity pathway analysis (IPA) was employed to identify the main pathways, biological processes, molecular functions, and cellular component associated with SARS-CoV-2 infection. The canonical pathways involved in the host response mainly related to immune response, inflammation, and coagulation. The chord diagrams in Figure 6A,B report the top 10 pathways and their relative proteins involved in critical (6A) and non-critical (6B) patients. The main pathways involved in non-critical and critical patients were the complement system pathway, acute-phase response signaling, the coagulation system, the LXR/RXR activation pathway, the extrinsic and intrinsic prothrombin activation pathway, the FXR/RXR activation pathway, IL-12 signaling and production in macrophages, the production of nitric oxide and reactive oxygen species in macrophages, and clathrin-mediated endocytosis signaling. As Figure 6C shows, some pathways were more altered in non-critical patients—specifically, acute phase response signaling, LXR/RXR, and FXR/RXR activation. Meanwhile, other pathways—such as the complement system, the coagulation system, and the extrinsic and intrinsic prothrombin activation pathway—were more altered in critical patients.

Diseases and disorders analysis highlighted the involvement of the inflammatory response and immunological disease, while physiological system development and function elaboration showed that the protein cargo is associated with a humoral immune response and immune cell trafficking (Figure 6D and Figure 6E).

### 2.6 Upstream Analysis Highlighted the Relation Between Inflammation and Protein Cargo

**FIGURE 7:**
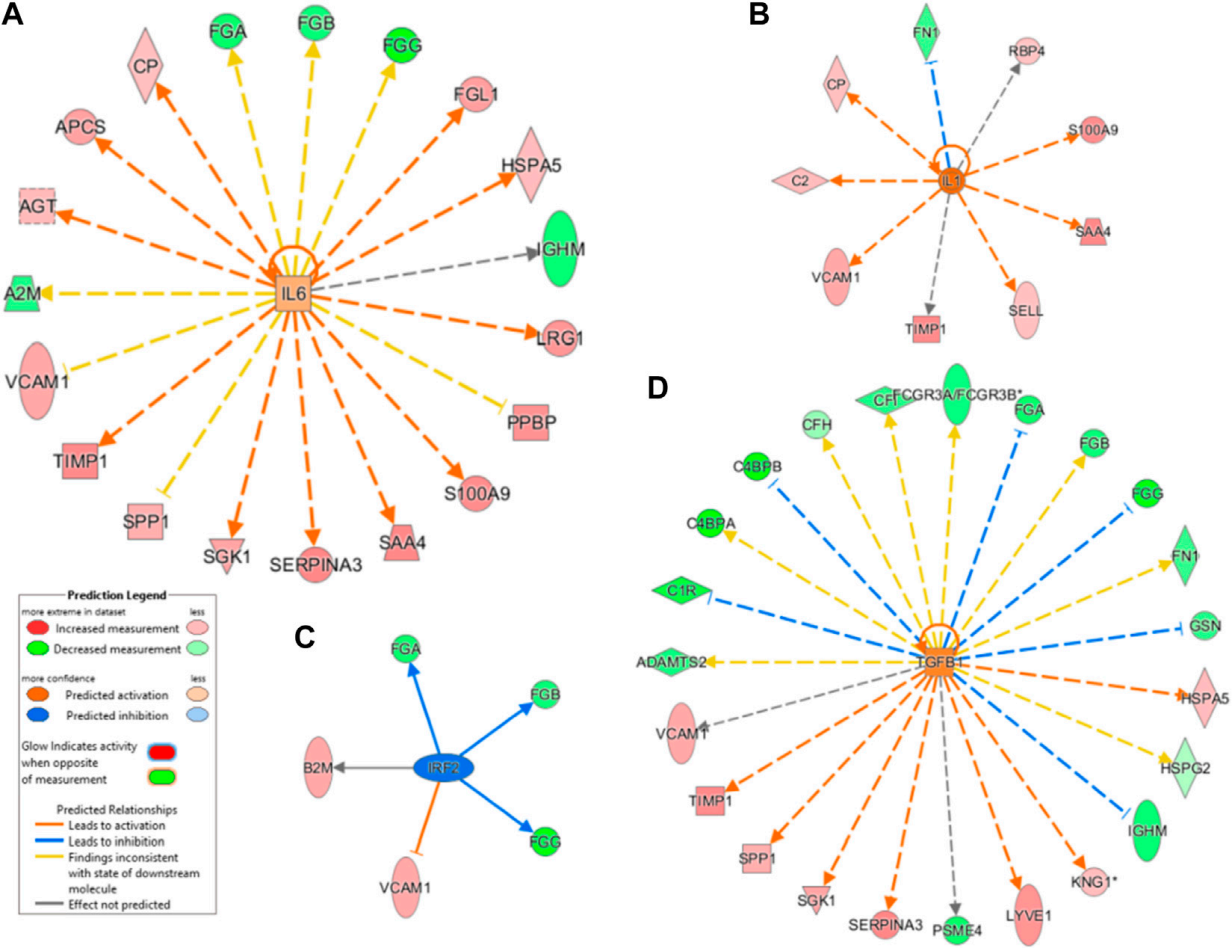
Canonical pathway analysis. A Chord diagram presenting a pathway analysis of significantly altered proteins in response to SARS-CoV-2 infection in critical **(A)** and non-critical **(B)** patients. Each pathway’s width is determined by the number of proteins associated with each pathway. Canonical pathways’ significance (-log(*p*-value)) was also compared **(C)**: this analysis showed similar exosome responses in critical and non-critical patients. The diseases and disorders analysis **(D)**, molecular and cellular functions **(E)**, and physiological system development and function elaboration **(F)** are also shown.

To predict the upstream molecules (transcription factor, microRNA, etc.) that could play a role in the observed proteome modulation and, thus, in the host response to SARS-CoV-2 infection, we performed upstream regulator analysis through IPA software. IPA analysis suggested that interleukin IL-6 (IL-6) and transforming growth factor (TGF)-beta1 (TGFB1) are the most significant upstream regulators (Figure 7A and Figure 7C). Among other upstream regulators, IL-1 was marked as a significantly activated regulator (z score=2.6) while IRF2 was predicted as an inhibited regulator (z score=-2.0), as Figure 7B,D show.

The dysregulation of IL-6–targeted genes (FGA, FGB, FGG, FGL1, HSPA5, IGHM, LRG1, PPBP, S100A9, SAA4, SERPINA3, SGK1, SPP1, TIMP1, VCAM1, A2M, AGT, APCS, and CP) and of TGFB1-targeted genes (VCAM1, TIMP1, SPP1, SGK1, SERPINA3, PSEM4, LYVE1, KNG1, IGHM, HSPG2, HSPA5, GSN, FN1, FGG, FGB, FGA, FCGR3A/FCGR3B, CP, CFH, C4BPB, C4BPA, C1R, and ADAMTS2) may contribute to SARS-CoV-2–related dysfunctions. As mentioned above, most of these regulated genes are involved in inflammation, immune response, and coagulation.

### 2.7 Association of Exosome Cargo with Pathologically Relevant Clinical Indices

We then evaluated whether exosomal protein cargo in COVID-19 patients significantly correlated with CRP and d-dimer levels, platelets, neutrophil, and monocytes counts. Spearman correlations were performed, and only correlations with p < 0.05 were considered and shown in Supplementary Table S5–S9. We found that circulating CRP levels positively correlated with CRP, IBP2, CHI3L1, FGB, FHR5, IGHV3-73, FGG, PRSS2, CFP, CFH, CD163, FCGBP, and CAT exosomal proteins, which are linked to inflammation, complement activation, and pulmonary fibrosis (Lu et al., 2011). Fibronectin, alpha-2-HS-glycoprotein, and alpha-1-acid glycoprotein 1 protein positively correlated with d-dimer levels, and platelet counts positively correlated with TIMP1, COL6A3, SPINK1, IGFBP4, IGHV1-8, NCAM1, COL18A1, APOA2, CFB, and MYH7 exosomal proteins, which are involved in platelets’ aggregation, adhesion, or activation and complement activation (Stecher et al., 1986).

Regarding neutrophil count, we found an increase in neutrophil among critical COVID-19 patients, and our analysis of exosomal cargo revealed a positive correlation with FGA protein, which is implicated in neutrophil activation (Rubel et al., 2001) but also with TPI1 protein, which has already been found in exosome cargo (Vargas et al., 2016), and with other inflammatory proteins (i.e., SAA1, coagulation factor XI, etc.).

Monocyte counts positively correlated with IGFALS, CFP, CLU, and SERPINC1 exosomal proteins, which are involved in the migration and chemotaxis of human monocytes (Peix et al., 2018).

### 2.8 Circulating Exosomes are Potential Biomarkers of COVID-19

**FIGURE 8:**
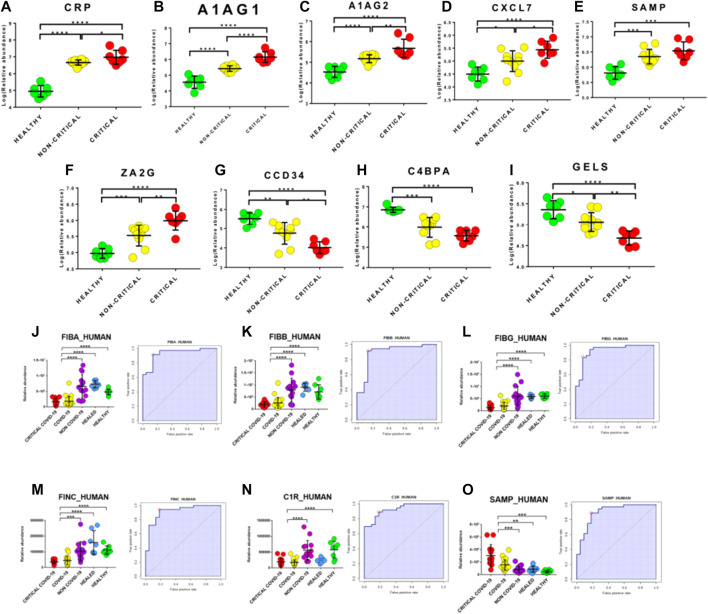
Upstream gene regulator analysis. IL-6 **(A)** and transforming growth factor (TGF)-beta1 **(B)** are the most significant upstream regulators. IL-1 resulted significantly activated (z score2.6) while interferon regulatory factor 2 was predicted as an inhibited regulator (z score-2.0).

Potential biomarkers were explored by carefully analyzing modulated proteins’ distribution and by using ROC curves. We firstly evaluated modulated proteins obtained from the discovery phase. Interestingly, we found that the abundance of several proteins directly correlated with the disease’s severity. In particular, critical patients displayed higher levels of CRP, A1AG1, A1AG2, CXCL7, SAMP, and ZA2G and lower levels of CCD34, C4BPA, and GELS than non-critical patients (Figures 8A–I). In addition, this analysis reported the presence of several proteins that are able to discriminate between COVID-19 patients and healthy subjects.

Biomarkers were validated on a new cohort of patients that included not only COVID-19 critical (n=13) and non-critical (n=26) patients and healthy subjects (n=7), but also critical (n=6) and non-critical (n=8) patients admitted for pneumonia and/or respiratory failure with negative nucleic acid test results (NON-COVID-19) and COVID-19 healed patients (n=7). Interestingly, fibrinogen proteins fibrinogen alpha chain (FIBA_HUMAN), fibrinogen beta chain (FIBB_HUMAN) and fibrinogen gamma chain (FIBG_HUMAN) showed an AUC value of 0.94 (sensitivity (SE): 86%; specificity (SP): 97%), 0.90 (sensitivity (SE): 92%; specificity (SP): 86%) and 0.93 (sensitivity (SE): 83%; specificity (SP): 91%), respectively. In addition, fibronectin (FINC_HUMAN), Complement C1r subcomponent (C1R_HUMAN) and Serum amyloid P-component (SAMP_HUMAN) showed an AUC value of 0.92 (sensitivity (SE): 94%; specificity (SP): 82%), 0.93 (sensitivity (SE): 89%; specificity (SP): 82%) and 0.91 (sensitivity (SE): 89%; specificity (SP): 82%), respectively (Figure 8J–O).

We also compared modulated exosomal proteins with regulated serum proteins in COVID-19 patients reported in the literature (D’Alessandro et al., 2020; Shen et al., 2020; Messner et al., 2020). Table 1 reports the list of common differentially expressed proteins. Interestingly, more than 50% of proteins (27 out of 50) presented the same direction of modulation identified in previous research on serum circulating proteins—even if the fold-change levels in plasma-exosome were higher than in serum.

**TABLE 1:** Comparison of modulated exosomal proteins with regulated serum proteins in COVID-19 patients reported in literature. Protein modulation (Up or Down) is indicated.

	Plasma-exosome		Serum (22)	Serum (23)	Serum (24)
Gene	Non-critical	Critical			
A1BG	Up	Up	−	−	Up
ACTB	−	Up	−	−	Up
AHSG	−	Up	Down	−	−
ALB	Up	−	Down	−	−
APO2	Up	−	−	Down	−
APOD	Up	−	−	Up	−
APOF	−	Up	Up	−	−
APOH	Down	Up	−	Down	−
C1R	Down	Down	−	−	Up
C1S	−	Down	−	−	Up
C4BPA	Down	Down	−	Up	−
C9	Up	−	Up	−	−
CLEC3B	−	Down	Down	−	−
CRP	Up	Up	Up	−	Up
CST3	−	Up	Up	−	−
F13B	Down	Down	Down	−	−
FCN2	Up	−	Down	Down	−
FGA	Down	Down	-	−	Up
FGB	Down	Down	-	−	Up
FGG	Down	Down	-	−	Up
FN1	Down	Down	-	−	−
GSN	Down	Down	Down	−	Down
HP	Up	−	−	−	Up
IGHV1-2	Down	−	Up	−	−
IGHV3-15	−	Down	Up	−	−
IGHV3–23	Down	Down	Up	−	−
IGHV3–9	Down	Down	Up	−	−
IGHV4–28	−	Down	Up	−	−
IGHV4–38–2	Down	−	Up	−	−
IGKV1–5	Down	−	Up	−	−
IGKV4–1	Down	−	Up	−	−
ITIH1	−	Down	Down	−	−
ITIH2	−	Down	Down	−	−
ITIH3	Up	−	Down	Up	Up
ITIH4	Up	−	Down	Up	Up
LRG1	Up	Up	Up	−	−
LYZ	−	Up	Up	−	−
ORM1	Up	Up	Up	Up	−
PGLYRP2	Down	−	Down	−	−
PON1	Up	−	Down	−	−
PPBP	Up	Up	Down	−	−
S100A8	−	Up	Up	−	−
S100A9	−	Up	Up	−	−
SAA4	Up	Up	−	Up	−
SERPINA3	Up	Up	Up	Up	−
SERPINA6	−	Up	Down	−	−
SERPING1	Up	Up	Up	−	−
TIMP1	Up	Up	Up	−	−
VCAM1	Up	Up	−	Up	−
VWF	−	Down	Up	−	−

## 3 Discussion

This study provides the first proteomic characterization of plasma-derived exosomes from COVID-19 patients and healthy controls. Participating patients were enrolled from a hospital located in Northern Italy, the first western epicenter of the COVID-19 pandemic. WBC, neutrophil, and eosinophil counts were increased in critical COVID-19 patients while the number of red blood cells, as well as lymphocytes, were significantly decreased (Terpos et al., 2020). Exosomes and EVs play significant roles in various biological functions and, particularly, in both physiological and pathological processes (Zhang et al., 2019). Indeed, they are associated with immune responses, viral pathogenicity, pregnancy, cardiovascular diseases, central nervous system–related diseases, and cancer progression (Kalluri and LeBleu, 2020).Several examples of scientific evidence have shown that viruses might use EVs to enter uninfected cells (Urbanelli et al., 2019). During the course of infections, EVs can convey pathogen molecules that serve as antigens or agonists of innate immune receptors to induce host defense and immunity or serve as regulators of host defense and mediators of immune evasion (Kadiu et al., 2012; Schorey and Harding, 2016).Our data reports, for the first time, the presence of viral material in COVID-19 patients’ host exosomal cargo. This finding suggests that SARS-CoV-2 may use the endocytosis route to spread infection throughout the host. We did not identify viral proteins via the purification of exosomes; thus, we can conclude that viral particles were not purified together with exosomes, suggesting that the RNA material was originally present in the cargo. A very recent study showed that exosomal microRNAs may drive thrombosis in COVID-19 patients (Gambardella et al., 2020) while Song and colleagues found that GM3-enriched exosomes positively correlated with disease severity, suggesting that they may participate in the pathological processes associated with COVID-19 progression (Song et al., 2020). Moreover, exosome-based strategies were also proposed to treat COVID-19 (Hassanpour et al., 2020) or prevent SARS-CoV-2 infection (Cocozza et al., 2020).

Our findings show that circulating exosomes are strongly involved in the processes associated with SARS-CoV-2 infection. Interestingly, our proteomic analysis of plasma-derived exosomes from COVID-19 patients revealed a specific proteomic signature. This signature was particularly evident using multivariate statistical analysis (PCA and PLS-DA), which highlighted the presence of proteomic features that are able to clearly discriminate between the samples, according to the diagnosis. Bioinformatics analysis revealed the presence of proteins related to the coagulation process, transport activity, complement activity, protease inhibitor activity, and defense/immunity protein activity.

**FIGURE 9:**
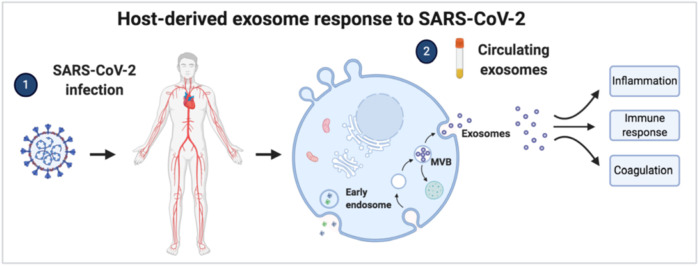
Box-plots and ROC curves for the best potential biomarkers identified using proteomic analysis of the exosome cargo. For **(A–I)**, box-plots of proteins that are well correlated with the disease’s severity are reported. Red dots indicate critical COVID-19 patients while yellow dots indicate non-critical COVID-19 patients. Fibrinogen alpha chain **(J)**, fibrinogen beta chain **(K)**, fibrinogen gamma chain **(L)** fibronectin **(M)**, Complement C1r subcomponent **(N)** and Serum amyloid P-component **(O)** were confirmed as good biomarkers in the validation. Purple dots indicate NON-COVID-19 patients while green and blue dots and indicate healthy and healed subjects, respectively.

Interestingly, exosomal proteins’ relative abundance in COVID-19 patients significantly differed from healthy subjects. Indeed, 157 and 97 proteins were significantly modulated in critical and non-critical COVID-19 patients, respectively. Our canonical pathway analysis performed on modulated proteins revealed the involvement of pathways associated with immune response, coagulation, and inflammation, as in Figure 9 summarizes.

The complement pathway is a double-edged sword for our immune system: it may offer protection by favoring viral clearance, but unrestrained activation may also result in pathological acute and chronic inflammations, tissue injury, and activation of the coagulation pathway (Noris et al., 2020). Complement activation has been linked to the pathophysiology of ARDS caused by various underlying diseases, and it has also been associated with COVID-19 (Risitano et al., 2020). Chahar et al. demonstrated that exosomes derived from respiratory syncytial virus–infected cells were able to activate an innate immune response by inducing cytokine and chemokine release from human monocytes and airway epithelial cells (Chahar et al., 2018). As Figure 6A,B show, we found a dysregulation of 17 proteins (C1QB, C1QC, C1R, C1S, C2, C3, C4A/C4B, C4BPA, C4BPB, C5, C6, C8A, C8G, CFI, CR1, MBL2, SERPING1) in critical patients associated with the complement system pathway while, in non-critical patients, 11 proteins were modulated (C1R, C4A/C4B, C4BPA, C4BPB, C8A, C8G, C9, CFH, CFI, MBL2, and SERPING1). In addition, the complement system pathway appeared more altered in critical patients (-log (p-value)=27.6) than non-critical patients (-log (p-value)=17.7). Among complement-related proteins, CFH and C4BPA had already been identified as transcriptional markers associated with severe SARS-CoV-2 infection: these molecules play central roles in complement activation and innate immunity (Ramlall et al., 2020). Moreover, we found an 2.5-fold increase of C9 in non-critical patients and a down-regulation of CFH; these findings suggest that, in critical COVID-19 patients, the C9 complement component might be impaired due to SARS-CoV-2 infection, as has already been shown for the hepatitis C virus (Kim et al., 2013). Interestingly, a global down-regulation of proteins coding for antibodies such as IGHV1-2, IGHV3-15, IGHV3-23, IGHV3-9, IGHV4-28, IGHV4-38-2, IGKV1-5, and IGKV4-1 was especially found in critical COVID-19 patients, suggesting the involvement of the humoral immune response, as Rolla et al. (2020) have already shown in reporting a significantly lower count of antibody-synthesizing lymphocyte among critical COVID-19 patients. Our results provide the first evidence of circulating exosomes’ potential immunomodulatory contribution in response to SARS-CoV-2 infection.

Furthermore, our analysis highlighted the implication of proteins in the acute-phase response pathway. The human immune system plays a key role in the defense against pathogens (Li et al., 2020). The acute-phase response is an innate body defense activated during acute illnesses, and it involves increased production of acute-phase proteins such as CRP and cytokines. In the case of an infection, the inflammatory response stimulates the liver to synthesize and release anti-inflammatory proteins that suppress inflammation and regulate immunity in the body (Gelain and Bonsembiante, 2019). As already reported, SARS-CoV-2 infected patients show high levels of pro-inflammatory cytokines and chemokines associated with pulmonary inflammation and extensive lung involvement; such inflammation has also been observed in SARS and MERS patients (Coperchini et al., 2020). Other studies have reported an elevation in acute-phase reactants among patients with COVID-19, including ESR, C-reactive protein (CRP), serum amyloid A, and ferritin—suggesting a rapid activation of the innate immune response (Jamilloux et al., 2020). Moreover, serum changes in acute-phase response proteins were used as indicators to identify the degree and progression of COVID-19. A significant increase in CRP and SAA content and a decrease C3 and C4 content have been associated with the disease’s severity (Li and Chen, 2020). In our study, among acute-phase proteins, exosomal CRP resulted in 122-fold and 40-fold up-regulation in critical and non-critical patients, respectively. A preliminary analysis of hematological and inflammatory parameters was associated with elevated CRP, IL-6, and NLR values and with worse outcomes as well as a lack of response to treatment (Montesarchio et al., 2020; Khinda et al., 2020). High C-reactive protein independently predicted the risk of mortality in a cohort of 183 COVID-19 patients (Rolla et al., 2020). Another study identified increased CRP levels in a limited number (31.0%) of COVID-19 patients (Ren et al., 2020). While high CRP levels were already reported during inflammation processes, our results indicate that CRP can be transported to other cells through EVs, thus driving the infection’s inflammatory effect. Alpha-1-acid glycoprotein protein (AGP), which is one of the major acute-phase proteins in humans, was up-regulated in both critical and non-critical patients. During the acute-phase response, the serum concentration of AGP increases as a liver hepatocytes response to the cytokines that are released by monocytes and other cells in the early phase of inflammation or infection (van Dijk et al., 1991). Alpha 1-antichymotrypsin (AACT_HUMAN) is another acute-phase protein that was found to be overexpressed in critical COVID-19 patients.

IL-12 signaling and production in macrophages pathways were significantly altered in both critical and non-critical patients, although we did not identify IL-12 protein directly in exosome cargo. Experimental evidence has shown that IL-12 rapidly activates both innate and specific immune responses, promoting host cellular responses, the clearance of the virus, and host recovery from infection (Komastu et al., 1998).

Interestingly, we found a significant modulation of the MSP-RON signaling pathway. This pathway contributes to the macrophage-induced immune response in order to assist the host in viral recognition via the macrophage stimulating protein (MSP) and the transmembrane receptor kinase RON Protein Tyrosine Kinase/Receptor d.

Coagulation’s involvement in SARS-CoV-2 infection has been extensively reported in COVID-19 (Palumbo et al., 2020; Al-Samkari et al., 2020). Proteins involved in platelet degranulation were down-regulated in SARS-CoV-2–infected patients (Zheng et al., 2020), together with the low platelet count associated with severe COVID-19 and mortality (Lippi et al., 2020; Shen et al., 2020). Our bioinformatics analysis demonstrated that exosomal proteins are associated with the coagulation system pathway and with the intrinsic and extrinsic prothrombin activation pathways. Complement factors are able to increase tissue factor activity, form activated thrombin from prothrombin, increase platelet activity and aggregation, increase prothrombinase activity, and release platelet-derived procoagulant granules (Fletcher-Sandersjöö and Bellander, 2020).

Among the effects of the viral ACE2 used by SARS-CoV-2 for cell entry, the connection between ACE2 and the Kallikrein/Kinin system—which regulates coagulation, fibrinolysis, and complement cascade—has resulted in great interest (Colarusso et al., 2020). Indeed, ACE2 physiologically binds and cleaves Lys-des-Arg9-Bradykinin and Des-Arg9 Bradykinin, which are potent ligands of Bradykynin Receptor 1 in the lungs (van de Veerdonk et al., 2020). When the virus blocks ACE2, the degradation of bradykinin cannot be performed, causing the accumulation of bradykinin itself; consequently, bradykinin—a product of high-molecular-weight kininogen—binds to its receptor (bradikynin-1receptor), triggering acute inflammation in the lungs due to the release of pro-inflammatory chemokines and cytokines such as the well-known IL-6 (Colarusso et al., 2020). Moreover, the kallikrein/kinin and renin-angiotensin systems also play a thrombo-regulatory role (Fang and Schmaier, 2020). Our data showed a two-fold up-regulation of Kininogen-1 in the exosomes of COVID-19 patients. Kininogen-1 itself has a relationship with the coagulation cascade, and it is considered an anti-thrombotic target. Indeed, its gene deletion has been associated with a reduced risk of induced thrombosis in mice (Fang and Schmaier, 2020). Hemostatic and thrombotic manifestations are common in critical COVID-19 patients, and they are among the leading causes of death (Al-Samkari et al., 2020 52), while a high number of venous thromboembolism (VTE) events among severely ill patients with COVID-19 pneumonia has been observed worldwide (Fontana et al., 2020). Relying on the role of Kininogen-1 and our findings, we speculate that exosomes may serve as a reserve and carrier of Kininogen-1.

Bioinformatic analysis has also pointed out the IL-6 and TGFβ1 cytokines’ involvement as upstream regulators of modulated protein. IL-6 is recognized as the main mediator of the inflammatory and immune response initiated by SARS-CoV-2 infection (Grifoni et al., 2020). As an upstream regulator of the exosomal proteins in COVID-19 patients, we speculate that IL-6 may also affect protein secretion from cells through EVs. TGFβ1 is a pleiotropic cytokine with regulatory capabilities, and it is involved in the resolution of the inflammatory response (Sanjabi et al., 2009). The host response to infection could be regulated by TGFβ1 with the help of a cytokine storm and the presence of TNF, IL-1β, and IL-6 (Chen, 2020). Lung fibrosis is among the clinical hallmarks of COVID-19 infection; indeed it was identified as a leading cause of pulmonary damage, as reported by Polak et al., (2020), who showed that 22% of patients presented a fibrotic pattern of lung injury characterized by interstitial fibrosis. Our data also suggest a correlation between TGFβ1, glucose, and exosomes. Interestingly, a correlation between a high concentration of glucose and worsening COVID-19 has been reported; for example, diabetic patients with uncontrolled glucose levels are evidently more prone to manifesting COVID-19 complications and consequent increased mortality—though the molecular mechanism currently remains undetermined (Zhu et al., 2020). Prolonged and uncontrolled hyperglycemia was also described as a prognostic factor (Brufsky, 2020). Most importantly, exosomes’ key role was already reported in the pathogenesis of diabetes nephropathy: high glucose leads to increased excretion of exosomes from macrophages through the TGF-β1 mRNA, which acts as a bridge between macrophages and mesangial cells (Zhu et al., 2019). A similar connection might also be applied to COVID-19, between macrophages and lung cells, even if other research is needed to investigate this hypothesis.

We also investigated exosomal proteins’ potential role as diagnostic biomarkers for COVID-19 infection and disease severity. Several exosomal proteins such as CRP, A1AG1, A1AG2, CXCL7, SAMP, and ZA2G proteins directly correlated with the disease’s severity, as their increase among critical patients showed, by suggesting a potential use of these proteins for monitoring the disease’s outcome and, potentially, any response to therapies. In line with our hypothesis, the exosomal proteome was already investigated to monitor sepsis progression (Xu et al., 2018) and HIV patients subjected to antiretroviral therapy (Chettimada et al., 2018). Huan et al. found that COVID-19 patients had significantly increased SAA and CRP levels, suggesting that SAA could serve as a biomarker to monitor the respiratory diseases’ progression (Huan et al., 2020). CRP levels positively correlated with the diameter of lung lesions and severe presentation (Wang, 2020; Matsumoto et al., 2019) but also with an increased risk of organ failure and death (Pierrakos and Vincent, 2010). Another interesting protein that we found to be up-regulated in critical patients is Fetuin-A. Exosomal Fetuin-A was already identified as a novel urinary biomarker for detecting acute kidney injury. We speculate that its up-regulation in critical COVID-19 patients can explain kidney injury, which is often reported in severe patients (Zhou et al., 2006).

Interestingly, the validation reported the presence of very good biomarker candidates—such as fibrinogen alpha chain, fibrinogen beta chain, fibrinogen gamma chain, fibronectin, complement C1r subcomponent and serum amyloid P-component—reporting an AUC from 0.94 to 0.91 with very good AUC values (from 0.94 to 0.91).

Intriguingly, all fibrinogen components in plasma exosomes were down-regulated in COVID-19 patients, suggesting an altered coagulation activity that could reflect a compensatory response to potential thrombotic manifestations.

Further, among the exosomal proteins modulated in SARS-CoV-2 infection we found LYVE1, TIMP1 and CXCL7 (Figure 5C). This expression profile supports to some extent the role of macrophage activation syndrome (MAS) as the main driver of hyperinflammatory response in COVID-19 patients (Schulert and Grom, 2015). Indeed, while LYVE-1-expressing macrophages control arterial stiffness through modulation of the metalloproteinase-dependent proteolysis of the MMP-9 matrix (Lim et al., 2018), the expression of the MMPs inhibitor TIMP1 is related to the frequency of pulmonary macrophages and is involved in influenza-induced lung injury (Allen et al., 2018) and CXCL7 is a known attractant of macrophages in lung inflammation (Unver et al., 2015).

In addition, our data revealed the presence of several exosomal proteins that present the same expression pattern as proteins detected in the serum of COVID-19 patients. Serum proteomics is usually performed on circulating serum proteins, with a bias not to discriminate between proteins contained in the exosome cargo used for cell-to-cell communication, and it may represent a picture of the mechanisms involved in COVID-19. Exosomes’ role may be particularly relevant in COVID-19 because SARS-CoV-2 infection is associated with tissue damage and multiple organ dysfunctions, and circulating exosomes can potentially reach several distant target cells and organs.

Taken together, our study’s findings provide the first evidence that circulating exosomes are strongly modulated during COVID-19 infection and might be involved in pathogenesis. The presence of viral material in the exosomal cargo showed that SARS-CoV-2 could use the cell-to-cell communication system to spread infection in the host. Proteomic analysis of plasma exosomes identified several molecules involved in immune response, inflammation, and the activation of coagulation and complement pathways, suggesting a significant role for exosomes in the mechanisms associated with tissue damage and multiple organ dysfunctions typical of COVID-19. Another remarkable result emerging from these data is the presence of several potential biomarkers that are well correlated with the disease’s severity. Although, to date, ours is the first study that characterizes the circulating exosomal proteins and pathways from SARS-CoV-2 infected patients, future studies are needed to determine the number and size of EVs in COVID-19. Moreover, monitoring exosomal content during infection may contribute to a better understanding of whether exosomes support viral spreading or induce immunological protection.

## 4 Materials and methods

### 4.1 Patients

For the discovery phase plasma samples from 17 subjects, admitted to Novara University Hospital for pneumonia and/or respiratory failure from March to April 2020 were collected at the Emergency Department or at COVID-19 wards including the Intensive Care Unit. All the patients had a confirmed diagnosis of SARS-CoV-2 infection by reverse-transcriptase polymerase chain reaction (RT-PCR).

We considered critical patients those with respiratory failure admitted to the intensive care unit requiring mechanical ventilation, while non-critical patients all other patients with mild to severe respiratory failure requiring oxygen supplementation but neither invasive nor noninvasive mechanical ventilation. All the patients were treated with Lopinavir / Ritonavir (Kaletra) and Hydroxychloroquine.

Out of the 17 COVID-19 patients enrolled, 7 were critical and 10 non-critical. Healthy individuals (n=7) were enrolled as controls. For the validation phase 36 COVID-19 patients, including non-critical (23) and critical (13) subjects, and on 39 non-COVID-19 patients, including 6 critical patients, 8 non-critical patients, 7 healthy subjects and 7 healed COVID-19 subjects were used. The Institutional Review Board (Comitato Etico Interaziendale Novara) approved this study. Clinical characteristics of the patients involved in the study are reported in Tables 2, 3.

**TABLE 2:** Characteristics of the patients included in the discovery phase study.

Variable	Non-COVID-19 patients	COVID-19 patients		
	Healthy Control (N=7)	Total (N=17)	Non-critical (N=10)	Critical (N=7)
Sex (no.)				
Male	2	8	5	3
Female	5	9	5	4
Age (year)				
Mean ± SD	51.4 ± 4.8	64.3 ± 16.5	68.3 ± 19.7	58.7 ± 8.8
Range	43.0 − 56.0	37.0 − 97.0	37.0 − 97.0	47.0 − 70.0
Time from onset to admission (days)				
Mean ± SD		5.2 ± 5.0	6.2 ± 5.8	3.6 ± 3.5
Range		1.0 − 18	1.0 − 18.0	1.0 − 9.0
Time from admission to severe (days)				
Mean ± SD				3.8 ± 2.9
Range				1.0 − 8.0
Symptoms (n°)				
Fever		8	4	4
Cough		6	5	2
Headache		0	0	0
Fatigue		1	1	0
Dyspnea		3	2	1
Diarrhea		2	1	1
Chest pain		1	1	0
Abdominal pain		1	0	1
Vomiting		1	1	0
Comorbidity (n°)				
Hypertension		4	1	3
Diabetes		6	2	4
Respiratory system		9	6	3
Cardiovascular system		6	3	3
Other endocrine system		2	0	2
Chronic kidney		3	0	3
Digestive system		4	1	3
Oxygen saturation index (%)				
Mean ± SD		93.4 ± 7.1	94.3 ± 4.4	92.1 ± 10.5
Range		71.0 − 99.0	86.0 − 99.0	71.0 − 98.0

**TABLE 3:** Characteristics of the patients included in the validation phase study.

Variables	COVID-19			Non-COVID-19				
Sex–no.	Total (N=36)	Non-Critical (N=23)	Critical (N=13)	Total (N=29)	Non-Critical (N=8)	Critical (N=6)	Healthy Control (N=8)	Healed (N=7)
Male	21	13	8	12	5	3	2	2
Female	15	10	5	17	3	3	6	5
Age-year								
Mean ± SD	65.6 ±17.6	66.5 ± 20.6	53.8 ± 10.7	60.4 ± 17.0	76 ± 12.9	68.6 ± 8.9	50.4 ± 5.4	46.8 ± 17.6
Range	35.0 − 101.0	35.0 − 101.0	49.0 − 84.0	30.0 − 96.0	59.0 − 96.0	56.0 − 82.0	43.0 − 56.0	30.0 − 72.0
Time from Onset to Admission, Days								
Mean ± SD	4.3 ± 4.5	4.5 ± 5.0	4 ± 3.6	4.8 ± 6.1	5.7 ± 7.0	1.0 ± 0.0		
Range	1.0 − 19.0	1.0 − 19.0	1.0 − 10.0	1.0 − 21.0	1.0 − 21.0	1.0 − 1.0		
Time from Admission to Severe, Days								
Mean ± SD			3.1 ± 2.8			3.0 ± 4.9		
Range			1.0 − 8.0			1.0 − 13.0		
Symptoms–no.								
Fever	18	11	7	3	1	2		
Cough	16	11	5	3	0	3		
Headache	0	0	0	2	0	2		
fatigue	3	2	1	3	0	3		
Dyspnea	8	6	2	5	1	4		
Diarrhea	4	2	2	0	0	0		
Chest pain	1	1	0	2	0	2		
Abdomial Pain	2	1	1	0	0	0		
vomit	1	1	0	1	1	0		
Comorbility–no.								
Hypertension	10	5	5	6	4	2		
Diabetes	8	4	4	1	0	1		
Respiratory system	4	2	2	1	1	0		
Cardiovascolar system	16	8	8	1	0	1		
Other Endocrine system	1	0	1	8	8	0		
Chronic Kidney	3	1	2	1	1	0		
Oxygen saturation index–%								
Mean ± SD	91.3 ± 7.4	93.3 ± 5.2	97.4 ± 9.6	92.1 ± 5.6	94.0 ± 4.1	85.5 ± 6.4		
Range	71.0 − 99.0	81.0 − 99.0	71.0 − 98.0	81.0 − 99.0	87.0 − 99.0	81.0 − 90.0		

### 4.2 Isolation of Plasma Exosomes

Exosomes were isolated using Exo-Spin exosome purification kit for plasma (Cell Guidance Systems, UK). In brief, 250 μl of plasma samples were centrifuged first at 300 g and then at 16000 g for 10 and 30 min, respectively, to remove platelets and larger vesicles. Half the volume of Exo-Spin buffer was added to the plasma samples, which were then mixed by inverting and incubated at 4°C for 1 h before centrifugation at 16 000 g for 60 min. Exosome pellets were resuspended in 100 μl phosphate-buffered saline (PBS) and purified using the Exo-Spin column. Finally, exosome were eluted in 200 μl PBS.

### 4.3 Nanoparticle Tracking Analysis (NanoSight NS300)

Particle size and concentration of plasma-derived exosomes were analysed by NTA using the NanoSight Technology NS300. In brief, exosomes were diluted in sterile saline buffer solution (1:100) and analyzed by the Nanoparticle Analyses System using the NTA 1.4 Analytical Software.

### 4.4 Western Blotting

Analysis of exosomes by immunoblotting was performed using standard protocols: proteins were denatured, separated on 4–12% polyacrylamide gels, transferred onto a nitrocellulose membrane and probed with antibodies against tetraspanins CD9 (Santa Cruz Biotechnology) and CD63 (Santa Cruz Biotechnology). The immunocomplexes were visualized by chemiluminescence using the Chemidoc MP imaging system (Bio-Rad Laboratories). Signal intensity of the bands was measured by using Image Lab software (Bio-Rad Laboratories).

### 4.5 One-Step Reverse Transcription-Droplet digital Polymerase Chain Reaction (RT-ddPCR)

Total RNA was extracted from 50 µl of plasma-derived exosomes using NucleoZOL (Macherey-Nagel) following manufacturer’s instruction.

SARS-CoV-2 RNA was quantified by means of the QX200TM Droplet Digital TM PCR System (ddPCR, Biorad) using the Bio-Rad SARS-CoV-2 ddPCR Kit and following manufacturer’s instruction. Data were analyzed using the QuantaSoftTM 1.7.4 Software (Bio-Rad) and SARS-CoV-2 quantification was expressed in number copies/10 µl of exosomes.

### 4.6 Immunodepletion of Ligh-Abundant Plasma Proteins and Digestion

Exosomes were lysed using 200 μl of RIPA buffer (50 mm Tris HCl pH 7.2, 0.05%SDS) and sonication. Proteins were then precipitated overnight using cold acetone at −20°C. The pellet was then resuspended using urea buffer and ammonium bicarbonate. In order to improve the identification and quantification of exosomal proteins we depleted high-abundance proteins using the Seppro IgY14 spin column kit (Sigma-Aldrich Inc., St. Louis, MO, USA) according to the manufacturer's procedure. The method was used to bind human serum HSA, IgG, fibrinogen, transferrin, IgA, IgM, haptoglobin, alpha 2-macroglobulin, alpha 1-acid glycoprotein, alpha 1-antitrypsin, Apo A-I HDL, Apo A-II HDL, complement C3 and LDL (ApoB) in order to increase low-abundance protein identification. The samples were transferred into an Amicon Ultra-0.5 mL 3 kDa centrifugal filter (Millipore, Billerica, MA, United States) following the manufacturer's procedure to collect high molecular weight proteins. The samples were then subjected to reduction with DTT 200 mM, to alkylation with IAM 200 mM and to complete protein digestion with 2 μg of Trypsin (Sigma-Aldrich Inc., St. Louis, MO, United States). The peptide digests were desalted on the Discovery® DSC-18 solid phase extraction (SPE) 96-well plate (25 mg/well) (Sigma-Aldrich Inc., St. Louis, MO, United States). The SPE plate was preconditioned with 1 mL of acetonitrile and 2 mL of water. After loading the sample, the SPE was washed with 1 mL of water. The adsorbed proteins were eluted with 800 μl of acetonitrile:water (80:20). After the desalting process, the sample was vacuum-evaporated and reconstituted in mobile phase for the analysis.

### 4.7 Proteomics Analysis and Data Processing

The digested peptides were analyzed with an EASY nano-LC 1200 system (Thermo Scientific, Milano, Italy) coupled to a 5600+ TripleTOF system (AB Sciex, Concord, Canada). The liquid chromatography parameters were as follows: analytical column Acclaim PepMap C18 2 μm 75µm x 150 mm and injection volume 2 μl. The flow rate was 300 nl/min, phase A was 0.1% formic acid/water and phase B was 80% acetonitrile/0.1% formic acid/20% water. A two hours gradient was used (3–45%). For identification purposes the mass spectrometer analysis was performed using a mass range of 100–1600 Da (TOF scan with an accumulation time of 0.25 s), followed by a MS/MS product ion scan from 400 to 1250 Da (accumulation time of 5.0 ms) with the abundance threshold set at 30 cps (40 candidate ions can be monitored during every cycle). The ion source parameters in electrospray positive mode were set as follows: curtain gas (N2) at 30 psig, nebulizer gas GAS1 at 25 psig, ionspray floating voltage (ISFV) at 2700 V, source temperature at 90°C and declustering potential at 85 V.

For label-free quantification, samples were then subjected to cyclic data independent analysis (DIA) of the mass spectra, using a 25-Da window. A 50 ms survey scan (TOF-MS) was performed, followed by MS/MS experiments on all precursors. These MS/MS experiments were performed in a cyclic manner using an accumulation time of 40 ms per 25-Da swath (36 swaths in total) for a total cycle time of 1.5408 s. The ions were fragmented for each MS/MS experiment in the collision cell using the rolling collision energy. The MS data were acquired with Analyst TF 1.7 (SCIEX, Concord, Canada).

The validation phase was performed using a micro-LC Eksigent Technologies (Dublin, United States) system with a stationary phase of a Halo Fused C18 column (0.5 × 100 mm, 2.7 μm; Eksigent Technologies, Dublin, United States). The mobile phase was a mixture of 0.1% (v/v) formic acid in water (A) and 0.1% (v/v) formic acid in acetonitrile (B), eluting at a flow-rate of 15.0 μl min −1 at an increasing concentration of solvent B from 2 to 40% in 30 min. Samples used to generate the SWATH-MS (Sequential window acquisition of all theoretical mass spectra) spectral library were subjected to the traditional data-dependent acquisition (DDA): the mass spectrometer analysis was performed using a mass range of 100-500 Da (TOF scan with an accumulation time of 0.25 s), followed by a MS/MS product ion scan from 200 to 1250 Da (accumulation time of 5.0 ms) with the abundance threshold set at 30 cps (35 candidate ions can be monitored during every cycle). Samples were then subjected to cyclic data independent analysis (DIA) of the mass spectra, using a 25-Da window. A 50 ms survey scan (TOF-MS) was performed, followed by MS/MS experiments on all precursors. These MS/MS experiments were performed in a cyclic manner using an accumulation time of 40 ms per 25-Da swath (36 swaths in total) for a total cycle time of 1.5408 s. The ions were fragmented for each MS/MS experiment in the collision cell using the rolling collision energy. The MS data were acquired with Analyst TF 1.7 (SCIEX, Concord, Canada).

The mass spectrometry files were searched using Protein Pilot (AB SCIEX, Concord, Canada) and Mascot (Matrix Science Inc., Boston, United States). Samples were input in the Protein Pilot software v. 4.2 (AB SCIEX, Concord, Canada), with the following parameters: cysteine alkylation, digestion by trypsin, no special factors and False Discovery Rate at 1%. The UniProt Swiss-Prot reviewed database containing human proteins (version 01/02/2018, containing 42271 sequence entries) and SARS-CoV-2 (version 28/04/2020, containing 13175 sequence entries) The Mascot search was performed on Mascot v. 2.4, the digestion enzyme selected was trypsin, with 2 missed cleavages and a search tolerance of 50 ppm was specified for the peptide mass tolerance, and 0.1 Da for the MS/MS tolerance. The charges of the peptides to search for were set to 2 +, 3 + and 4 +, and the search was set on monoisotopic mass. The instrument was set to ESI-QUAD-TOF and the following modifications were specified for the search: carbamidomethyl cysteines as fixed modification and oxidized methionine as variable modification (Dalla Pozza et al., 2017; Dalle Carbonare et al., 2018).

The quantification was performed by integrating the extracted ion chromatogram of all the unique ions for a given peptide. The quantification was carried out with PeakView 2.2 and MarkerView 1.2. (Sciex, Concord, ON, Canada). Six peptides per protein and six transitions per peptide were extracted from the SWATH files. Shared peptides were excluded as well as peptides with modifications. Peptides with FDR lower than 1.0% were exported in MarkerView for the t-test.

Statistical analysis and related graphical representations were done using GraphPad Prism v. 8 and MetaboAnalyst software (www.metaboanalyst.org). Ingenuity Pathways Analysis (IPA) software (Qiagen, Redwood City, CA, United States) and FunRich (http://www.funrich.org) were used for bioinformatics analysis.

## Data Availability

The datasets G for this study can be found in the proteomexchange database Project accession: PXD021144.
