# Supplementary material for: Circulating Exosomes Are Strongly Involved in SARS-CoV-2 Infection
Source: Front Mol Biosci. 2021 Feb 22;8:632290. doi: 10.3389/fmolb.2021.632290 (PMC7937875; doi:10.3389/fmolb.2021.632290)
Supplement: Supplementary file 2 [file table2.docx]

**FIGURES**

**Fig. 1**: Overview of this study’s experimental design: plasma exosomes from 10 non-critical COVID-19 patients, seven critical COVID-19 patients, and seven healthy subjects were isolated using a commercial kit. The exosomes’ protein content was analyzed using proteomics analysis (nanoLC-HRMS), and the identified and modulated proteins were elaborated with bioinformatics in order to identify the host-derived exosome response to SARS-CoV-2 and its associated pathways. The analysis suggested the presence of new biomarkers. The validation of potential exosomal biomarkers was performed on an external cohort of patients using a proteomics approach on a microLC-HRMS. 36 COVID-19 patients, including non-critical (23) and critical (13) subjects, and on 28 non-COVID-19 patients, including 6 critical patients, 8 non-critical patients, 7 healthy subjects and 7 healed COVID-19 subjects were analyzed.

**Fig. 2**: Plasma-exosomes protein content. A Venn diagram (A) of identified proteins in critical and non-critical COVID-19 patients and in healthy subjects. Gene ontology classification of identified proteins based on body component (B), molecular function (C), and biological process (D) was also used to assess the functions associated to identified proteins.

**Fig. 3**: Principal component analysis. The score plot (A) of PC1 and PC2 shows a clear separation of healthy subjects (green dots) from critical COVID-19 patients (red dots) and non-critical (yellow dots) COVID-19 patients. (B) The bi-plot of the scores (black) and loading (red) of PC1 and PC2 reported the correlation between the severity of the disease and the expression of proteins.

**Fig. 4**: A partial least square discriminant analysis (PLS-DA) of critical patients (red dots), non-critical patients (yellow dots), and healthy subjects (green dots). The three groups are well separated (A). Important features identified by PLS-DA (B): colored boxes indicate the most predictive or discriminative features in each group (red, high; blue, low; white, middle). A dendrogram from the hierarchical clustering (C) of protein abundance in the exosomes from critical patients (red), non-critical patients (yellow), and healthy subjects (green).

**Fig. 5**: Modulated exosomal proteins in SARS-CoV-2 infection. Volcano plots of quantified proteins (A and B) reporting p-value and fold change. A total of 157 and 97 proteins were modulated in critical (A) and non-critical (B) COVID-19 patients, respectively (p-value < 0.05 and fold change > 1.3). Hierarchical heat maps of quantified proteins (C) highlighting the three clusters of samples, with critical COVID-19 patients in red, non-critical COVID-19 patients in yellow, and healthy subjects in green.

**Fig. 6**: Canonical pathway analysis. A Chord diagram presenting a pathway analysis of significantly altered proteins in response to SARS-CoV-2 infection in critical (A) and non-critical (B) patients. Each pathway’s width is determined by the number of proteins associated with each pathway. Canonical pathways’ significance (-log(p-value)) was also compared (C): this analysis showed similar exosome responses in critical and non-critical patients. The diseases and disorders analysis (D), molecular and cellular functions (E), and physiological system development and function elaboration (F) are also shown.

**Fig. 7**: Upstream gene regulator analysis. IL-6 (A) and transforming growth factor (TGF)-beta1 (B) are the most significant upstream regulators. IL-1 resulted significantly activated (z score=2.6) while interferon regulatory factor 2 was predicted as an inhibited regulator (z score=-2.0).

**Fig. 8**: Box-plots and ROC curves for the best potential biomarkers identified using proteomic analysis of the exosome cargo. For 8A to 8I, box-plots of proteins that are well correlated with the disease’s severity are reported. Red dots indicate critical COVID-19 patients while yellow dots indicate non-critical COVID-19 patients. Fibrinogen alpha chain (J), fibrinogen beta chain (K), fibrinogen gamma chain (L) fibronectin (M), Complement C1r subcomponent (N) and Serum amyloid P-component (O) were confirmed as good biomarkers in the validation. Purple dots indicate NON-COVID-19 patients while green and blue dots and indicate healthy and healed subjects, respectively.

**Fig. 9**: Host-derived exosome response to SARS-CoV-2 infection. Circulating exosomes are characterized by proteins and pathways involved in inflammation, immune response, and coagulation.
