# Supplementary material for: Circulating Exosomes Are Strongly Involved in SARS-CoV-2 Infection
Source: Front Mol Biosci. 2021 Feb 22;8:632290. doi: 10.3389/fmolb.2021.632290 (PMC7937875; doi:10.3389/fmolb.2021.632290)
Supplement: Supplementary file 3 [file table3.docx]

**Supplementary data**

**Supplementary figure 1**: Characterization of exosomes isolated from the plasma of healthy patients. (A) Western blot analysis of the expression of proteins enriched in exosomes, including CD63 and CD9. (B) Nanoparticle tracking analysis to quantify the size and concentration of exosomes.

**Supplementary Table 1**: clinical indices of critical and non-critical COVID-19 patients.

|  | **Critical**  **(n=7)** | **Non-critical**  **(n=10)** | ***p-value*** |
| --- | --- | --- | --- |
| D-dimer ng/mL | 1927.40(1137-3030) | 1127.37(298-2799) | 0.12 |
| Circulating CRP mg/L | 14.22(2.84-32.21) | 5.96(0.38-15.82) | 0.073 |
| PLTs (*10^3^/µl) | 305.57(45-514) | 207.40(33-350) | 0.13 |
| **RBCs (*10^3^/µl)** | **3.18(2.79-3.99)** | **4.53(2.67-6.07)** | **0.0071** |
| **WBC (*10^3^/µl)** | **13.52(6.48-23.05)** | **6.80(1.75-14.11)** | **0.017** |
| Neutrophils (*10^2^/µl) | 81.88(65.6-93.1) | 69.51(47.7-89.8) | 0.063 |
| **Lymphocytes (*10^2^/µl)** | **11.04(3.9-19.4)** | **23.18(6.7-46)** | **0.034** |
| Monocytes (*10^2^/µl) | 5.14(2.2-11) | 6.59(2.6-9.8) | 0.32 |
| **Eosinophils (*10^2^/µl)** | **1.55(0-4.6)** | **0.28(0-1.2)** | **0.027** |
| Basophils (*10^2^/µl) | 0.37(0.1-1.1) | 0.44(0-1.7) | 0.75 |

**Supplementary Table 2**: identified proteins in critical and non-critical COVID-19 patients (xlsx file).

**Supplementary Table 3**: list of modulated proteins in critical COVID-19 patients (xlsx file).

**Supplementary Table 4**: list of modulated proteins in non-critical COVID-19 patients (xlsx file).

**Supplementary Table 5**: Correlation analysis between proteins abundance and platelets count. The p-value, R square and r are reported for the most correlated proteins.

| **Accession name** | **Protein name** | **Gene** | **P (two-tailed)** | **R squared** | **r** |
| --- | --- | --- | --- | --- | --- |
| TIMP2_HUMAN | Metalloproteinase inhibitor 2 | TIMP2 | 0,010 | 0,36 | 0,60 |
| K2C6A_HUMAN | Keratin, type II cytoskeletal 6A | KRT6A | 0,011 | 0,35 | -0,59 |
| ANXA6_HUMAN | Annexin A6 | ANXA6 | 0,014 | 0,33 | 0,58 |
| CO6A3_HUMAN | Collagen alpha-3(VI) chain | COL6A3 | 0,014 | 0,33 | 0,57 |
| ISK1_HUMAN | Serine protease inhibitor Kazal-typ**e** | SPINK1 | 0,016 | 0,32 | 0,57 |
| IBP4_HUMAN | Insulin-like growth factor-binding protein | IGFBP4 | 0,020 | 0,30 | 0,55 |
| IBP2_HUMAN | Insulin-like growth factor-binding protein | IGFBP2 | 0,022 | 0,30 | 0,55 |
| HV108_HUMAN | Immunoglobulin heavy variable 1-8 | IGHV1-8 | 0,022 | 0,30 | 0,54 |
| VSIG4_HUMAN | V-set and immunoglobulin domain | VSIG4 | 0,025 | 0,28 | 0,53 |
| NCAM1_HUMAN | Neural cell adhesion molecule 1 | NCAM1 | 0,027 | 0,28 | -0,53 |
| COIA1_HUMAN | Collagen alpha-1(XVIII) chain | COL18A1 | 0,032 | 0,26 | 0,51 |
| APOA2_HUMAN | Apolipoprotein A-II | APOA2 | 0,033 | 0,26 | -0,51 |
| APOC2_HUMAN | Apolipoprotein C-II | APOC2 | 0,034 | 0,26 | -0,51 |
| CFAB_HUMAN | Complement factor B | CFB BF | 0,035 | 0,26 | 0,51 |
| MYH7_HUMAN | Myosin-7 | MYH7 | 0,039 | 0,25 | 0,50 |
| K2C75_HUMAN | Keratin, type II cytoskeletal 75 | KRT75 | 0,043 | 0,24 | -0,49 |
| CH3L1_HUMAN | Chitinase-3-like protein 1 | CHI3L1 | 0,048 | 0,23 | 0,48 |

**Supplementary Table 6**: Correlation analysis between proteins abundance and circulating CRP levels. The p-value, R square and r are reported for the most correlated proteins.

| **Accession name** | **Protein name** | **Gene** | **P (two-tailed)** | **R squared** | **r** |
| --- | --- | --- | --- | --- | --- |
| CRP_HUMAN | C-reactive protein | CRP | 0,0002 | 0,69 | 0,83 |
| IBP2_HUMAN | Insulin-like growth factor-binding protein | IGFBP2 | 0,0003 | 0,67 | 0,82 |
| CH3L1_HUMAN | Chitinase-3-like protein 1 | CHI3L1 | 0,0005 | 0,65 | 0,80 |
| MOONR_HUMAN | Protein moonraker | KIAA0753 | 0,0007 | 0,63 | 0,79 |
| ISK1_HUMAN | Serine protease inhibitor Kazal-type | SPINK1 | 0,0011 | 0,60 | 0,77 |
| IBP4_HUMAN | Insulin-like growth factor-binding protein | IGFBP4 | 0,0023 | 0,55 | 0,74 |
| FIBB_HUMAN | Fibrinogen beta chain | FGB | 0,0028 | 0,53 | 0,73 |
| FHR5_HUMAN | Complement factor H-related protein | CFHR5 | 0,0028 | 0,53 | 0,73 |
| HV373_HUMAN | Immunoglobulin heavy variable 3-73 | IGHV3-73 | 0,0082 | 0,45 | 0,67 |
| FIBG_HUMAN | Fibrinogen gamma chain | FGG | 0,009 | 0,44 | 0,66 |
| TRY2_HUMAN | Trypsin-2 | PRSS2 | 0,014 | 0,40 | 0,63 |
| LYVE1_HUMAN | Lymphatic vessel endothelial hyaluronic | LYVE1 | 0,016 | 0,39 | 0,62 |
| PROP_HUMAN | Properdin | CFP | 0,016 | 0,39 | 0,62 |
| FIBA_HUMAN | Fibrinogen alpha chain | FGA | 0,019 | 0,37 | 0,61 |
| APC_HUMAN | Adenomatous polyposis coli protein | APC | 0,031 | 0,33 | -0,57 |
| CFAH_HUMAN | Complement factor H | CFH | 0,033 | 0,32 | 0,57 |
| C163A_HUMAN | Scavenger receptor cysteine-rich type | CD163 | 0,035 | 0,31 | 0,56 |
| FCGBP_HUMAN | IgGFc-binding protein | FCGBP | 0,036 | 0,31 | 0,56 |
| CATA_HUMAN | Catalase | CAT | 0,042 | 0,30 | 0,54 |

**Supplementary Table 7**: Correlation analysis between proteins abundance and D-dimer levels. The p-value, R square and r are reported for the most correlated proteins.

| **Accession name** | **Protein name** | **Gene** | **P (two-tailed)** | **R squared** | **r** |
| --- | --- | --- | --- | --- | --- |
| FETUA_HUMAN | Alpha-2-HS-glycoprotein | AHSG | 0,0053 | 0,52 | 0,72 |
| KPYM_HUMAN | Pyruvate kinase PKM | PKM | 0,007 | 0,49 | 0,70 |
| FINC_HUMAN | Fibronectin | FN1 | 0,014 | 0,43 | -0,65 |
| A1AG1_HUMAN | Alpha-1-acid glycoprotein 1 | ORM1 | 0,022 | 0,38 | 0,62 |
| VSIG4_HUMAN | V-set and immunoglobulin domain | VSIG4 | 0,025 | 0,53 | -0,73 |
| TRY6_HUMAN | Putative trypsin-6 | PRSS3P2 | 0,041 | 0,46 | -0,68 |
| SAMP_HUMAN | Serum amyloid P-component | APCS | 0,041 | 0,32 | -0,57 |
| MOONR_HUMAN | Protein moonraker | KIAA0753 | 0,044 | 0,46 | -0,67 |
| CO4B_HUMAN | Complement C4-B | C4B | 0,044 | 0,31 | -0,56 |

**Supplementary Table 8**: Correlation analysis between proteins abundance and neutrophils counts. The p-value, R square and r are reported for the most correlated proteins.

| **Accession name** | **Protein name** | **Gene** | **P (two-tailed)** | **R squared** | **r** |
| --- | --- | --- | --- | --- | --- |
| FIBA_HUMAN | Fibrinogen alpha chain | FGA | 0.0051 | 0.41 | 0.64 |
| TPIS_HUMAN | Triosephosphate isomerase | TPI1 | 0.010 | 0.36 | -0.60 |
| IGLC7_HUMAN | Immunoglobulin lambda constant 7 | IGLC7 | 0.010 | 0.36 | -0.6 |
| CLUS_HUMAN | Clusterin | CLU | 0.012 | 0.34 | 0.58 |
| SAA1_HUMAN | Serum amyloid A-1 protein | SAA1 | 0.013 | 0.34 | 0.58 |
| APC_HUMAN | Adenomatous polyposis coli protein | APC | 0.016 | 0.32 | -0.57 |
| FA11_HUMAN | Coagulation factor XI | F11 | 0.017 | 0.32 | 0.56 |
| SAA2_HUMAN | Serum amyloid A-2 proteincur | SAA2 | 0.018 | 0.31 | 0.56 |
| QSOX1_HUMAN | Sulfhydryl oxidase 1 | QSOX1 | 0.027 | 0.28 | 0.53 |
| ANT3_HUMAN | Antithrombin-III | SERPI | 0.027 | 0.28 | 0.53 |
| PLAK_HUMAN | Junction plakoglobin | JUP | 0.030 | 0.27 | -0.52 |
| EMSY_HUMAN | BRCA2-interacting transcriptional | EMSY | 0.032 | 0.26 | -0.51 |
| NUCL_HUMAN | Nucleolin | NCL | 0.036 | 0.26 | 0.51 |
| TRY3_HUMAN | Trypsin-3 | PRSS3 | 0.039 | 0.25 | -0.50 |
| ANXA6_HUMAN | Annexin A6 | ANXA6 | 0.040 | 0.25 | 0.50 |
| IGHG4_HUMAN | Immunoglobulin heavy constant gamma | IGHG4 | 0.040 | 0.25 | 0.50 |
| ZA2G_HUMAN | Zinc-alpha-2-glycoprotein | AZGP1 | 0.041 | 0.24 | 0.49 |
| TRY6_HUMAN | Putative trypsin-6 | PRSS3P2 | 0.042 | 0.24 | -0.49 |
| CO8A_HUMAN | Complement component C8 alpha chain | C8A | 0.046 | 0.23 | 0.48 |

**Supplementary Table 9**: Correlation analysis between proteins abundance and monocytes counts. The p-value, R square and r are reported for the most correlated proteins.

| **Accession name** | **Protein name** | **Gene** | **P (two-tailed)** | **R squared** | **r** |
| --- | --- | --- | --- | --- | --- |
| ALS_HUMAN | Insulin-like growth factor-binding | IGFALS | 0.0032 | 0.45 | -0.67 |
| PROP_HUMAN | Properdin | CFP | 0.0049 | 0.41 | 0.64 |
| CLUS_HUMAN | Clusterin | CLU | 0.0071 | 0.39 | -0.62 |
| ZA2G_HUMAN | Zinc-alpha-2-glycoprotein | AZGP1 | 0.011 | 0.35 | -0.59 |
| ANT3_HUMAN | Antithrombin-III | SERPINC1 | 0.013 | 0.34 | -0.58 |
| FA11_HUMAN | Coagulation factor XI | F11 | 0.018 | 0.31 | -0.56 |
| QSOX1_HUMAN | Sulfhydryl oxidase 1 | QSOX1 | 0.027 | 0.28 | -0.53 |
| HV373_HUMAN | Immunoglobulin heavy variable 3-73 | IGHV3-73 | 0.028 | 0.28 | 0.52 |
| GPX3_HUMAN | Glutathione peroxidase 3 | GPX3 | 0.034 | 0.26 | -0.51 |
| TPM4_HUMAN | Tropomyosin alpha-4 chain | TPM4 | 0.037 | 0.25 | -0.50 |
| A2GL_HUMAN | Leucine-rich alpha-2-glycoprotein | LRG1 | 0.042 | 0.24 | -0.49 |
| APOC3_HUMAN | Apolipoprotein C-III | APOC3 | 0.044 | 0.24 | -0.49 |
| AACT_HUMAN | Alpha-1-antichymotrypsin | SERPINA3 | 0.044 | 0.24 | -0.49 |
| SEPP1_HUMAN | Selenoprotein P | SELENOP | 0.047 | 0.23 | -0.48 |
| IFFO2_HUMAN | Intermediate filament family orphan | IFFO2 | 0.048 | 0.23 | 0.48 |
